# A comparison of tobacco product prevalence by different frequency of use thresholds across three US surveys

**DOI:** 10.1186/s12889-021-11283-w

**Published:** 2021-06-24

**Authors:** Luz María Sánchez-Romero, Christopher J. Cadham, Jana L. Hirschtick, Delvon T. Mattingly, Beomyoung Cho, Nancy L. Fleischer, Andrew Brouwer, Ritesh Mistry, Stephanie R. Land, Jihyoun Jeon, Rafael Meza, David T. Levy

**Affiliations:** 1 Department of Oncology, Lombardi Comprehensive Cancer Center, Georgetown University Medical Center, 3970 Reservoir Road NW, Washington, USA; 2 Department of Epidemiology, University of Michigan School of Public Health, 1415 Washington Heights, Ann Arbor, MI USA; 3 Department of Health Behavior and Health Education, University of Michigan School of Public Health, 1415 Washington Heights, Ann Arbor, MI USA; 4 Tobacco Control Research Branch, National Cancer Institute, 9609 Medical Center Blvd., Bethesda, MD USA

**Keywords:** Tobacco products, Prevalence, Surveys and questionnaires, United States

## Abstract

**Background:**

With the increasing changes in tobacco use patterns, “current use” definition and the survey used may have important implications for monitoring population use trends.

**Methods:**

Using three US surveys (2014/15 TUS-CPS, NHIS and PATH), we compared the adult (age 18+) prevalence of four product groups (cigarettes, other combustibles, smokeless tobacco, and e-cigarettes) based on three past 30-day frequency of use thresholds: 1+, 10+, and 25+ days. We also examined mutually exclusive single, dual, and polytobacco users as a percentage of total users for each product group.

**Results:**

Regardless of threshold or product, the prevalence was higher in PATH followed by NHIS and TUS-CPS, in some cases by large percentages. The differences in cigarette and smokeless tobacco use prevalence in going from the 1+ to 10+ days and to the 25+ days threshold were minimal. Applying different frequency thresholds had the largest impact on other combustibles prevalence, with a 60% reduction with the 10+ days threshold and a 80% reduction with the 25+ days threshold, compared to the 1+ days threshold, followed by e-cigarettes with 40 and 60% reductions, respectively. The proportion of dual and polytobacco users decreased considerably when using the 10+ vs. the 1+ days threshold and polytobacco use was almost non-existent with the 25+ days threshold.

**Conclusion:**

The estimated prevalence of each tobacco product use depends largely on the survey and frequency of use threshold adopted. The choice of survey and frequency threshold merits serious consideration when monitoring patterns of tobacco use.

**Supplementary Information:**

The online version contains supplementary material available at 10.1186/s12889-021-11283-w.

## Background

With tobacco use as the leading preventable cause of death [1], measures of use prevalence play a central role in monitoring the health of the population. Estimates of prevalence help determine use trends, assess the impact of tobacco control policies, and identify high-risk groups. These estimates depend on the definition of tobacco use for different types of tobacco.

In the US, current adult cigarette use prevalence is generally defined in terms of whether an individual consumed more than 100-cigarettes in their lifetime (established use), combined with whether the individual now smokes every day or some days [2, 3]. However, some surveys, such as the Global Adult Tobacco Survey [4], do not include a question on whether the individual smoked 100-lifetime cigarettes, as this criterion may not adequately reflect patterns of usage for some countries, age groups, or ethnicities [5, 6]. In addition, someday users (individuals who have smoked 100-cigarettes or more in their lifetime and who do not smoke every day) are a heterogeneous group [7, 8]. Therefore, for some studies or public health purposes, it may be important to go beyond the someday use classification and distinguish between frequent and infrequent someday smokers based on a threshold of the number of days smoked in the past month.

While cigarette smoking remains a central concern, the reduction in smoking prevalence [9] in the United States (US) in recent years has been accompanied by an increase in the use of e-cigarettes [10] and other non-cigarette tobacco products (e.g. smokeless tobacco) [11–14]. In estimating the use of other non-cigarette tobacco products (e.g., e-cigs, cigars, cigarillos, snus), current use is often defined as use on at least one day in the past 30 days [15] or every day or someday [16]. While this measure is likely to be most inclusive, it may not adequately distinguish regular patterns of use from sporadic, short-term, or experimental use [17, 18]. With increases in use of non-cigarette tobacco products both in terms of exclusive and multiple product (dual; concurrent use of two products and polytobacco; concurrent use of 3+ products) use, particularly in some population subgroups, the choice of frequency of use threshold (minimum number of days used to be considered a frequent user) appropriate for each product’s use pattern may have important implications for monitoring some trends and behaviors. For example, the US National Youth Tobacco Survey has defined frequent use of a tobacco product as using a product on ≥20 days of the past 30 days [19]. In addition, prevalence estimates may depend on the survey considered. Differences in surveys aim, methodology, sample size and questions asked may affect estimates of product use prevalence [20, 21], and have important implications for surveillance.

In this study, our aim is to examine how different frequency of use thresholds and choice of survey impact prevalence estimates of current adult tobacco product use. We estimated the prevalence of four groups of tobacco products (cigarettes, other combustibles, smokeless tobacco and e-cigarettes) based on three past 30-day frequency of use thresholds across three US nationally representative surveys. We also considered how the prevalence of exclusive, dual and polytobacco differs as a function of frequency threshold. We compared three surveys: the Tobacco Use Supplement to the Current Population Survey (TUS-CPS), the National Health Interview Survey (NHIS), and the Population Assessment of Tobacco and Health (PATH). The NHIS is the principal source of information on the health of the civilian noninstitutionalized population of the United States and is used for evaluating health oriented goals, such as Healthy People 2020 [22]. The TUS-CPS is a larger survey often used for estimating socio-demographic breakdowns and PATH is a longitudinal survey that assesses population tobacco use and health.

## Methods

### Surveys

We used data from three nationally representative US adult (ages 18 and above) surveys that include tobacco use questions and were temporally comparable: the 2014/15 TUS-CPS, the 2015 NHIS and Wave 2 (2014/15) of the PATH, The 2014/15 TUS-CPS used Computer Assisted Telephone Interviewing (CATI) or Computer Assisted Personal Interviewing (CAPI) for data collection [23, 24]. The 2015 NHIS used CAPI [25]. Wave 2 of PATH (2014/15) collected data using CAPI and Audio Computer-Assisted Self-Interviewing (ACASI) [26]. We focused on the years 2014/15 because all surveys had data available for that year and collect data for each of the different tobacco products. In addition, we considered that use patterns, particularly for e-cigarettes, had stabilized (i.e., after the rise in 2014 and before the dramatic increase in Juul use beginning in 2017) [21]. Further details on the surveys’ methods and the specific questions asked for each product category and tobacco product grouping are provided in Supplementary Tables 1 and 2.

### Measurement

For this study, we classified tobacco products into four groups: (a) cigarettes, (b) other combustibles (traditional cigars, cigarillos, filtered cigars, tobacco pipe, and hookah), (c) smokeless tobacco (snus, dissolvable tobacco and other smokeless tobacco) and (d) e-cigarettes (e-cigarettes, vape-pens, hookah pens, e-hookahs or e-vaporizers). For current *cigarette users,* we applied the 100-cigarettes lifetime criterion since it is applied in all three surveys and is commonly used. No lifetime use criterion was applied to the remaining *tobacco products* (other combustibles, smokeless tobacco, and e-cigarettes). There is no established consensus on the cut-off to define levels of frequency of use [27–29]. For this study, to differentiate current use based on the frequency of use, we applied three different thresholds for the number of days used in the past 30 days: one or more (1+), ten or more (10+) and twenty five or more (25+) days. The 1+ day measure was chosen as it is the standard measure to define “current use” when assessing frequency of tobacco use, whereas the 10+ day measure was based on our previous work [21, 30]. The 25+ days threshold was chosen as a measure of most frequent established users, similar to daily use. When current use information was missing for any of the four product groups, the observation was omitted from the sample.

Additionally, we created a mutually exclusive 16 category patterns-of-use variable based on combinations of our four tobacco product groupings, including non-use (1 category), single (4 categories), dual (two product types, 6 categories) and poly (three or four product types, 5 categories) use (Supplementary Table 3). Respondents who were missing information on the category variable were excluded (0.3% PATH; 1.6% TUS-CPS; 6.3% NHIS). Dual and polyuse were defined as the use of multiple product groups using the same 1+, 10+, or 25+ days threshold. For example, a 10+ days dual user of cigarettes and e-cigarettes would have used both products on 10 or more days in the last month (i.e., the individuals reported consumption in 10+ days in the last 30 days for each separate tobacco product question). Respondents who reported everyday use were classified as having used on 30 out of the past 30 days. All respondents reporting no current use were coded as using 0 out of the past 30 days.

### Statistical analysis

For each survey, we calculated the weighted prevalence of current use using three frequency of use thresholds (1+ day, 10+ days and 25+ days in the past 30 days) after appropriately accounting for the complex survey design of each sample. We used the Balanced Repeated Replication variance estimation method with Fay’s adjustment set to 0.3 [31] and cross sectional weights for PATH, Taylor Series Linearization [32] for NHIS and replicated weights for TUS-CPS as recommended by the surveys. These analyses were conducted using Stata version 15 [33].

Because the overall prevalence of product use varied considerably across surveys, the prevalence estimates by frequency of use threshold depend on the initial level for a particular frequency measure. To make the change in estimates by frequency measure comparable across surveys, we estimated the relative difference in prevalence among tobacco users in each survey by using the 1+ vs. the 10+ days use thresholds and the 1+ vs. the 25+ days use thresholds. For example, the 10+ days measure is compared to the 1+ day measure using (10+ days - 1 + day)/1+ day. Similarly, we examined the prevalence of single, dual and polyusers in each survey as a percentage of total users (i.e., the sum of all mutually exclusive categories using data from Supplementary Table 4) for each of the four product groups, e.g., exclusive cigarette users as a percent of all exclusive and multiproduct cigarette users.

## Results

TUS-CPS had the largest analytic sample size after exclusions (*n* = 155,067), followed by NHIS (*n* = 31,709) and PATH (*n* = 28,070). The weighted distributions of sociodemographic characteristics were generally similar across surveys, with approximately 48% male and 70% aged 35 or older [34].

### Patterns of product use

Table 1 shows the weighted prevalence of current use based on three frequency of use measures and the relative difference between estimates for any users of each of the four tobacco product groups by survey. For each of the four product categories, estimates by threshold and differences between them across all surveys showed similar trends, a reduction in overall use of any product prevalence as frequency of use threshold increases, particularly from the 1+ days to 10+ days.

**Table 1:** Current use prevalence of four tobacco product groups in three surveys based on three frequency of use thresholds in the past 30 days: 1+ day, 10+ days, 25+ days

Products	National Surveys					
	TUS-CPS % (95% CI)		NHIS % (95% CI)		PATH % (95% CI)	
		RR*		RR*		RR*
**Cigarettes**						
1+ day	13.6 (13.4, 13.8)		14.9 (14.3, 15.4)		18.8 (18.3, 19.4)	
10+ days	12.7 (12.6, 12.9)	−6.6%	13.8 (13.2, 14.4)	−7.4%	17.0 (16.5, 17.5)	−9.6%
25+ days	11.0 (10.9, 11.2)	−19.1%	12.0 (11.5, 12.5)	−19.5%	15.2 (14.7, 15.7)	−19.1%
**Other combustibles**						
1+ day	2.1 (2.1, 2.2)		3.2 (2.9, 3.6)		5.7 (5.4, 5.9)	
10+ days	0.7 (0.7, 0.7)	− 66.7%	1.9 (1.7, 2.2)	−40.6%	1.6 (1.5, 1.7)	− 71.9%
25+ days	0.4 (0.4, 0.5)	− 81.0%	1.7 (1.5, 2.0)	− 46.9%	1.0 (0.9, 1.1)	− 82.5%
**Smokeless tobacco**						
1+ day	1.6 (1.5, 1.6)		2.1 (1.8, 2.4)		2.8 (2.6, 3.0)	
10+ days	1.3 (1.3, 1.4)	−18.8%	1.7 (1.5, 2.0)	−19.0%	2.2 (2.0, 2.4)	−21.4%
25+ days	1.1 (1.0, 1.1)	−31.3%	1.4 (1.2, 1.6)	−33.3%	1.8 (1.7, 2.0)	−35.7%
**E-cigarettes**						
1+ day	2.2 (2.1, 2.2)		3.1 (2.8, 3.4)		4.6 (4.3, 4.8)	
10+ days	1.4 (1.3, 1.4)	− 36.4%	2.0 (1.8, 2.2)	−35.5%	2.5 (2.3, 2.7)	− 45.7%
25+ days	0.9 (0.8, 0.9)	− 59.1%	1.3 (1.1, 1.5)	− 58.1%	1.8 (1.6, 2.0)	−60.9%

*Relative Reduction. Relative reduction 10+ days compared to 1+ day and 25+ days compared to 1+ day

Abbreviations: TUS-CPS Tobacco Use Supplement to the Current Population Survey; NHIS, National Health Interview Survey; PATH, Population Assessment of Tobacco and Health

#### Comparison of prevalence estimates across surveys

Compared to TUS-CPS cigarette use prevalence estimates (13.6% with the 1+ day, 12.7% with the 10+ days and 11.0% with the 25+ days thresholds), NHIS estimates were about 10% higher in relative terms and estimates from PATH were nearly 40% higher in relative terms (e.g., 1+ day frequency of cigarette use for PATH = 18.8% and TUS-CPS = 13.6%: 18.8–13.6%/13.6% = 38.2%) regardless of the threshold. The prevalence estimates for other combustibles from NHIS and PATH were more than double those from TUS-CPS (2.1% for 1+ day, 0.7% for 10 + days and 0.4% for 25+ days) regardless of the threshold. Compared to the smokeless tobacco prevalence from TUS-CPS (1.6% for 1 + day, 1.3% for 10+ days and 1.1% for 25+ days), NHIS use prevalence was 30% higher regardless of the threshold while PATH estimated prevalence for the 1+ day was 75% higher and 60% greater with the 10+ days or 25+ days thresholds. The e-cigarette use prevalence estimates from TUS-CPS (2.2% for 1 + day, 1.4% for 10+ days and 0.9% for 25+ days) were about 50% lower, in relative terms than estimates from NHIS regardless of the threshold, and PATH estimates for e-cigarette use were more than double estimates from TUS-CPS when using the 1+ day or 25+ days thresholds.

#### Relative differences in product use prevalence by frequency of use thresholds within surveys

Estimates from TUS-CPS showed that when compared to the 1+ day threshold, cigarette prevalence declined by 6.6% for the 10+ days and by 19.1% for the 25+ days thresholds, while smokeless tobacco prevalence declined by 18.8% for the 10 + days and by 31.3% for the 25+ days thresholds. For other combustibles, compared to the 1+ day frequency of use, the prevalence declined by 66.7% for the 10+ days and by 81.0% for the 25+ days thresholds, while e-cigarette use declined by 36.4% for the 10+ days and by 59.1% for the 25+ days thresholds. None of the 95% confidence intervals for prevalence estimates within a product category overlapped between different thresholds, suggesting important differences between estimated prevalence.

When comparing TUS-CPS estimates with those in NHIS and PATH, we observed that for cigarette use, the relative difference in prevalence between thresholds was similar when using either 10+ days or 25+ days vs. 1+ day thresholds although slightly greater in PATH (9.6% vs. 6.6% for TUS-CPS and 7.4% for NIHS) when using the 10+ days threshold. For other combustibles, relative differences in prevalence using 10+ days or 25+ days vs. 1+ day thresholds were generally lower in NHIS (about 40%) compared to TUS-CPS and PATH which had similar relative differences (about 70 and 80% respectively for both surveys). The relative differences between thresholds observed for smokeless tobacco and e-cigarettes were similar across all three surveys. Thus applying increasing use thresholds (1+ to 10+ to 25+ days) impacted use prevalence the least for cigarettes, followed by smokeless tobacco, and the most for other combustibles and e-cigarettes.

### Patterns of exclusive, dual and polytobacco use

In Table 2, using the 1+ day frequency of use threshold, TUS-CPS data showed that 83.4% of all cigarette users were exclusive users (i.e., 11.1%/13.3%, see Supplementary Table 4), increasing to 90.9% for the 10+ days and to 95.4% for the 25+ days thresholds. Slightly smaller proportions were estimated from NHIS. However, PATH proportion of exclusive use for the 1+ day threshold was considerably lower (70.5%) than the other surveys but similar proportions to NHIS were observed for the 10+ days (86.2%) and the 25+ days (92.3%) thresholds. In TUS-CPS, the percentage of dual use among any cigarette users for the 1+ day thresholds was 9.0% with e-cigarettes, 4.5% with other combustibles and 1.5% with smokeless tobacco. Using the 10+ days threshold, the proportions of dual use were reduced to 5.6% for e-cigarettes, 1.6% for other combustibles, 1.6% for smokeless, and to 2.8, 0.9 and 0.9% using the 25+ days threshold for each product respectively. The proportions of three or more products (polyuse) among any cigarette users showed larger reductions than exclusive and dual use when going from the 1+ to the 10+ days or the 25+ days threshold. In general, the percent of exclusive cigarette use prevalence increased with increasing frequency of use threshold, as dual and poly cigarette users moved into the exclusive use category with the 10+ and 25+ day measures.

**Table 2:** Percentage of exclusive, dual and polytobacco use as a proportion of any cigarette users by three frequency of use thresholds for three national surveys

Products (%)	TUS-CPS			NHIS			PATH		
	1+ day	10+ days	25+ days	1+ day	10+ days	25+ days	1+ day	10+ days	25+ days
**Exclusive Use**									
Cigarettes	83.4	90.9	95.4	79.3	87.4	92.2	70.5	86.2	92.3
**Dual Use of Cigarettes +**									
E-cigarettes	9.0	5.6	2.8	9.5	5.9	2.6	11.8	6.5	3.4
Other combustibles	4.5	1.6	0.9	6.1	4.4	4.3	10.2	4.2	2.7
Smokeless tobacco	1.5	1.6	0.9	2.7	1.5	0.9	2.7	2.4	1.3
**Poly Use of Cigarettes +**									
E-cigarettes + other combustibles	0.8	0.2	0.0	1.6	0.7	0.1	2.7	0.4	0.1
E-cigarettes + smokeless	0.3	0.1	0.0	0.3	0.1	NA	0.7	0.1	0.1
Other combustibles + smokeless	0.3	0.1	0.0	0.4	0.1	0.0	0.8	0.2	0.1
E-cigarettes+ other combustibles + smokeless	0.2	0.0	0.0	0.2	0.0	NA	0.5	0.1	0.0
*Total all polytobacco use (3 or 4 products)*	*1.5*	*0.3*	*0.1*	*2.7*	*0.7*	*0.2*	*4.8*	*0.7*	*0.3*

NA = no sample was available for this group. * 0.0 values are = < 0.01.

Abbreviations: TUS-CPS = Tobacco Use Supplement to the Current Population Survey; NHIS=National Health Interview Survey; PATH=Population Assessment of Tobacco and Health

Table 3 shows that in TUS-CPS, the percentage of exclusive use among all other combustible users was 55.6% for the 1+ day threshold increasing to 61.5% for the 10+ days and to 73.2% for the 25+ days thresholds, with similar estimates from NHIS. In contrast, PATH estimates were about 50% regardless of the frequency of use threshold. For TUS-CPS, the percentage of other combustible dual users with cigarettes was 27.8% for 1+ day, increasing to 30.8% for the 10+ days and decreasing to 24.4% for the 25+ days thresholds. NHIS showed similar patterns for all thresholds, while PATH showed proportionately greater increases from the 1 + day to the 10+ days threshold, but more stability in going from the 10+ days to the 25+ days threshold.

**Table 3:** Percentage of exclusive, dual and polytobacco use as a proportion of any other combustible users by three frequency of use thresholds for three national surveys

Products (%)	TUS-CPS			NHIS			PATH		
	1+ day	10+ days	25+ days	1+ day	10+ days	25+ days	1+ day	10+ days	25+ days
**Exclusive Use**									
Other combustibles	55.6	61.5	73.2	56.4	60.9	68.6	45.8	48.2	52.6
**Dual Use of Other combustibles +**									
Cigarettes	27.8	30.8	24.4	28.2	30.5	28.6	33.5	42.2	42.1
E-cigarette	4.6	1.5	2.4	3.1	2.0	1.1	5.3	1.8	1.1
Smokeless Tobacco	4.6	1.5	0.0	3.1	1.5	1.1	1.8	1.2	1.1
**Poly Use of Other combustible +**									
Cigarettes + E-cigarettes	4.6	3.1	0.0	6.3	4.6	0.6	8.8	3.6	1.1
Cigarettes + smokeless	1.9	1.5	0.0	1.9	0.5	0.0	2.6	1.8	1.1
E-cigarettes + smokeless	0.0	0.0	NA	0.0	NA	NA	0.5	0.6	1.1
Cigarettes + e-cigarettes + smokeless	0.9	0.0	0.0	0.9	0.0	NA	1.8	0.6	0.0
*Total all polytobacco use (3 or 4 products)*	*9.3*	*3.1*	*2.4*	*9.4*	*5.1*	*1.1*	*14.1*	*6.0*	*3.2*

NA = no sample was available for this group. * 0.0 values are ≤0.01.

Abbreviations: TUS-CPS = Tobacco Use Supplement to the Current Population Survey; NHIS=National Health Interview Survey; PATH=Population Assessment of Tobacco and Health

In Table 4, smokeless tobacco users showed a relatively high percentage of exclusive use although less than cigarettes, regardless of the threshold. Using TUS-CPS, the proportion of exclusive smokeless tobacco use was 75.0% using the 1+ day threshold, increasing to 81.5% for the 10+ days and to 90.9% for the 25+ days thresholds. In comparison, NHIS reported a lower percentage of exclusive use with the 1+ day threshold, but similar proportions for the 10+ days and 25+ days thresholds, while PATH showed lower proportions than NHIS and TUS-CPS regardless of the threshold. The most frequent dual use combination was with cigarettes followed by other combustibles. While the TUS-CPS proportion of dual use of smokeless tobacco with cigarettes increased in going from 1+ day to 10+ days threshold and declined with the 25+ days threshold, the proportion of dual use kept decreasing at higher thresholds in NHIS and PATH.

**Table 4:** Percentage of exclusive, dual and polytobacco use as a proportion of any smokeless tobacco users by three frequency of use thresholds for three national surveys

Products (%)	TUS-CPS			NHIS			PATH		
	1+ day	10+ days	25+ days	1+ day	10+ days	25+ days	1+ day	10+ days	25+ days
**Exclusive Use**									
Smokeless tobacco	75.0	81.5	90.9	69.0	83.8	90.9	62.7	76.2	85.6
**Dual Use of Smokeless Tobacco +**									
Cigarettes	12.5	14.8	9.1	19.7	12.0	7.6	18.5	17.9	10.7
E-cigarettes	0.0	1.5	0.0	0.0	1.2	0.0	0.0	1.8	1.1
Other-combustibles	6.3	0.7	0.0	4.9	1.8	1.5	3.7	0.9	0.5
**Poly Use of Smokeless Tobacco +**									
Cigarettes + e-cigarettes	2.5	0.7	0.0	2.0	0.6	NA	4.8	0.9	0.5
Cigarettes + other combustibles	2.5	0.7	0.0	3.0	0.6	0.0	5.5	1.3	1.1
E-cigarettes + other combustibles	0.0	0.0	NA	0.0	NA	NA	1.1	0.4	0.5
Cigarettes + e-cigarettes + other combustible	0 1.3	0.0	0.0	1.5	0.0	NA	3.7	0.4	0.0
*Total all polytobacco use (3 or 4 products) s*	*6.3*	*1.5*	*0.9*	*4.9*	*1.2*	*0.0*	*14.8*	*3.1*	*2.1*

NA = no sample was available for this group. * 0.0 values are ≤0.01.

Abbreviations: TUS-CPS = Tobacco Use Supplement to the Current Population Survey; NHIS=National Health Interview Survey; PATH=Population Assessment of Tobacco and Health

Table 5 shows that the percentage of exclusive use among all e-cigarette users from TUS-CPS was 32.0% using the 1+ day threshold increasing to 44.1% for the 10+ days and to 65.9% for the 25+ days thresholds. In general, all surveys showed proportions of exclusive e-cigarette users increasing with higher frequency thresholds, especially in going from 10+ days to 25+ days. Compared to TUS-CPS, higher proportions of exclusive e-cigarette users were estimated using NHIS for all three frequency of use thresholds. While PATH’s proportion of exclusive e-cigarette users was lower than TUS-CPS using the 1+ day threshold, it was higher with the 10+ days and the 25+ days thresholds. The TUS-CPS proportion of dual e-cigarette with cigarette use among all e-cigarette users, was 54.8% for the 1+ day, 51.5% for the 10+ days, and 33.0% for the 25+ days threshold, with similar patterns found in NHIS and PATH. Mixed patterns were observed for other dual combinations across all surveys, but large reductions were generally observed in using the 10+ vs. the 1+ day threshold. The proportion of polytobacco users (three or more product groups) among any e-cigarette users showed proportionately larger reductions with increasing frequency of use threshold than dual users.

Prevalence estimates for all 16 mutually exclusive categories can be found in Supplementary Table 4.

**Table 5:** Percentage of exclusive, dual and polytobacco use as a proportion of any e-cigarette users by three frequency of use thresholds for three national surveys

Products (%)	TUS-CPS			NHIS			PATH		
	1+ day	10+ days	25+ days	1+ day	10+ days	25+ days	1+ day	10+ days	25+ days
**Exclusive Use**									
E-cigarettes	32.0	44.1	65.9	41.4	51.0	73.2	28.2	48.6	68.2
**Dual Use of E-cigarettes +**									
Cigarette	54.8	51.5	33.0	44.6	40.8	24.4	47.7	44.5	28.4
Other combustibles	4.6	0.7	1.1	3.2	2.0	1.6	6.5	1.2	0.6
Smokeless tobacco	0.9	1.5	0.0	1.3	1.0	0.0	0.9	1.6	1.1
**Poly Use of E-cigarettes +**									
Cigarettes+ other combustibles	5.0	1.5	0.0	7.3	4.6	0.8	11.1	2.4	0.6
Cigarettes+ Smokeless	1.8	0.7	0.0	1.3	0.5	NA	2.8	0.8	0.6
Other combustibles + Smokeless	0.0	0.0	NA	0.0	NA	NA	0.7	0.4	0.6
Cigarettes+ other combustibles + smokeless	0.9	0.0	0.0	1.0	0.0	NA	2.2	0.4	0.0
*Total all polytobacco use (3 or 4 products)*	*8.2*	*2.2*	*1.1*	*9.6*	*4.6*	*0.8*	*16.7*	*4.0*	*1.7*

NA = no sample was available for this group. * 0.0 values are ≤0.01.

Abbreviations: TUS-CPS = Tobacco Use Supplement to the Current Population Survey; NHIS=National Health Interview Survey; PATH=Population Assessment of Tobacco and Health

## Discussion

Our analysis focused on the variation in current use prevalence estimates of four categories of tobacco products (cigarettes, other combustibles, smokeless tobacco and e-cigarettes) overall and distinguished by exclusive, dual and polyuse. We compared the estimated current use prevalence from the TUS-CPS, NHIS and PATH national surveys using three frequency of use thresholds (1+, 10+, and 25+ days in the past 30 days).

The relative differences in current use prevalence using the 1+ day compared to the 10+ days or the 25+ days thresholds were generally consistent across the three surveys. We observed that the prevalence reduction in current cigarette and smokeless tobacco users as the threshold increased was minimal (less than 20%), indicating that the frequency of use in the past 30 days for these users was relatively stable regardless of the threshold used. This lower variability for cigarette estimates might be due in part to the adoption of the “100-cigarette lifetime” criteria for established use. The largest variations were observed for other combustibles, followed by e-cigarettes (around 60%). Previous studies of tobacco product frequency of use [7, 8, 30, 35, 36] have generally observed important differences in prevalence within nondaily users. For example, a study of smokeless tobacco prevalence found that prevalence using a 1+ day threshold was about 17% higher than that using a 10+ days threshold, [30] and another study found that e-cigarette prevalence doubled when using a 1+ day compared to a 20+ days threshold [21].

In assessing multiproduct use, we found that the proportions of exclusive use for cigarettes and smokeless tobacco users tended to be 63% or higher, even when using the 1+ day threshold. In contrast, other combustibles and e-cigarette users were more inclined towards dual and less frequent use with the proportion of exclusive use of these products at around 30% with the 1+ day threshold, reaching 70% when using the 25+ days threshold. Other studies have also found relatively high rates of multiproduct use among e-cigarette and other combustible users, especially when examining use at low frequency thresholds [37–39].

In terms of the most appropriate threshold, we do not prescribe a particular measure, since the choice of threshold should depend on the purpose for which the definition is applied. In gauging some types of public health impacts, a stable measure of regular use is likely more appropriate. While cigarette and smokeless tobacco use was generally more frequent and stable, the large differences between the 1+ and the 10+ days thresholds for e-cigarettes and other combustibles suggest that these products have a less stable usage pattern, consistent with evidence that suggests these products are used more by experimental and social users [19]. In addition, e-cigarettes are the newest tobacco product, with use patterns still to be understood. Dual and polyuse prevalence rates also generally fell substantially when the threshold increased, signaling that multiproduct usage patterns may be less frequent and less stable. However, polyusers have shown greater nicotine dependence than single users [38, 40], suggesting a potential tendency towards future regular use. In addition, while more frequent use of tobacco products is generally associated with more harmful health effects than less frequent use, less frequent use may be relevant in terms of the pathways of usage of current users towards long-term pattern and also has important health implications [41–43]. In particular, one study found that lifelong non-daily smokers who reported 11–30 cigarettes per month had a 34% higher mortality risk compared to never smokers [41].

In determining patterns of initiation and transitions from experimental to regular use, a more sensitive measure of use might be more appropriate. Infrequent use may be particularly relevant in assessing transitions to regular use, especially among e-cigarette and other combustible users [44] but also among cigarette and smokeless tobacco users [45, 46]. Focusing on a lower frequency of use may also be especially important in capturing transitions in dual and polytobacco use patterns. For example, dual e-cigarette users have shown greater cessation intentions compared with exclusive cigarette smokers [47]. Also, cigarette smokers who use smokeless tobacco have been found to be more likely than exclusive smokers to attempt quitting cigarette smoking using other tobacco products [48].

In choosing the appropriate measure, different thresholds may be needed for different product categories. For e-cigarettes and other combustibles, lower thresholds (e.g.,1+ day) may be more relevant in estimating potential transitions between product use, and the 10+ days threshold may be more useful in assessing more frequent and stable use. To assess the relevance of different thresholds, it will be important to develop evidence on the stability of use and transitions over time using longitudinal data such as PATH.

Our results also suggest that the appropriate frequency measure may depend on the survey used. Comparing across surveys, the prevalence of current use was higher in PATH followed by NHIS and TUS-CPS, regardless of the product or frequency of use threshold. Despite using the standard definition of established cigarette use (i.e., the 100-cigarettes lifetime criteria), we observed inter-survey variability in cigarette prevalence estimates. There was also variability in the estimated smokeless tobacco prevalence. However, we observed greater variability in e-cigarettes and other combustibles use prevalence. Other studies comparing different surveys have also reported similar variations in current use prevalence for cigarettes [49], smokeless tobacco [50], and e-cigarettes [21]. Some of the variations across surveys may be due to differences in the current use definition (in previous studies) or the sampling procedure, method of interview, and design of each survey. For example, PATH has a longitudinal and more complex design than TUS-CPS and NHIS surveys and this cohort characteristic may be a limitation as population subgroups may change over time as respondents drop out from the survey. The failure to understand the variation in prevalence estimates across surveys is a gap in the literature [20, 51]. With the increasing use of convenience or crowdsourced surveys (e.g., Mechanical Turk) [52], it becomes important to validate the prevalence from these surveys against a larger nationally representative survey. However, our analysis suggests that the validation may depend on the survey used. It may also be important to consider trends over time in the measures used. For example, while we found substantial differences in the measure for 2015, trends over comparable periods may be similar across surveys. Nevertheless, changes in data collection questions within the same survey over the years should be considered in analyzing trends as it can make it difficult to compare prevalence over time.

### Limitations

Our results are subject to limitations. First, for the three frequency of use measures, we focused on 1+, 10+ and 25+ days out of the past 30 days, in order to consider potential measures of “experimenters”, “infrequent users” and “frequent users” across non-cigarette tobacco products. However, for cigarettes, we applied the 100-cigarettes lifetime criterion. The use of this criterion could explain in part the relative stability in smoking prevalence estimates when changing the frequency threshold, as the measure captures established users. Although not applied in this study, some surveys [53] have used or are using a lifetime criterion to define established users for other products; for cigars, use at least 50 times in their lifetime [54] and for smokeless tobacco use at least 20 times in their lifetime [55–57]. However, their use has yet to be standardized to all surveys. Further research is merited on the relevance of these criteria as they may apply to other products. Studies should further consider different frequency of use thresholds to determine whether these cut-offs are the most relevant. Other indicators of use, such as the duration of use, intensity and biomarkers [18, 44, 58] also merit attention. Second, we grouped nicotine delivery products into four classes to allow for comparisons between surveys. The wide variations in prevalence by different frequency of use thresholds found for the other combustibles category indicate that further exploration is warranted for products included within that category (i.e., little cigars, premium cigars, pipe, and hookah) [59, 60]. The frequency of use estimates of each of these products varies (e.g., hookah is less regularly used than cigars) by threshold. Other product groupings may be relevant depending on the purpose of the product use definition. Third, to estimate dual and polyuse prevalence, we applied the same frequency of use threshold (e.g., 10+ days) to the different products considered. However, combinations for dual and polyuse with varying frequency thresholds by product may have different stability and transitional properties. Fourth, we focused on the year 2015 due to availability of data for those three surveys and because use patterns seemed to be relatively stable compared to earlier and later years [21]. Consequently, our results do not reflect the increased prevalence of e-cigarette use in recent years. With the rapid evolution of e-cigarette use and the introduction of heated tobacco products into the US market, it will be important to consider the stability of use patterns over time. Finally, our results focus on the population as a whole. Different measures may be needed by age, gender, racial/ethnic, socioeconomic status, and mental health status to characterize high-risk groups. While not discussed in the results section, we considered age variations and found that the prevalence of the four types of tobacco products tended to be higher among 18–34 year-olds than ages 35+ when using the 1+ and 10+ days thresholds, but lower for the 25+ days. However, gender differences were less clear and no relevant differences between thresholds were observed.

## Conclusion

Our analysis indicates that tobacco product use prevalence is subject to a complex set of variations across frequency of use thresholds and surveys. Due to the heterogeneity of use patterns across surveys and within product groups, common definitions of *current use* (e.g., 100-cigarettes lifetime, or any use in the past 30 days) as a one-size-fits-all may not adequately address tobacco use patterns and their related public health implications. The appropriate measure will depend on the purpose. Different frequency of use thresholds for overall, dual and polytobacco use and variations across subpopulations and overtime may be needed to better capture recent product use patterns and their impact on public health.

## Supplementary Information

**Additional file 1 Supplementary Table 1.** Surveys Description. **Supplementary Table 2.** Questions used to collect data about the number of days use in the past 30 days for each of the three national health surveys. **Supplementary Table 3.** List of Mutually Exclusive Categories of single, dual and poly tobacco product use. **Supplementary Table 4.** Population prevalence of exclusive, dual, and polytobacco users using three frequency of use thresholds for three national surveys*.

## Data Availability

The datasets generated and/or analyzed during the current study are available from the Population Assessment of Tobacco and Health Study Public Use Files, https://www.icpsr.umich.edu/web/NAHDAP/studies/36498/datadocumentation The Tobacco Use Supplement to the Current Population Survey. https://cancercontrol.cancer.gov/brp/tcrb/tus-cps/questionnaires-data The National Health and Interview Survey. https://www.cdc.gov/nchs/nhis/nhis_2014_data_release.htm
