# Supplementary material for: A comparison of tobacco product prevalence by different frequency of use thresholds across three US surveys
Source: BMC Public Health. 2021 Jun 24;21:1203. doi: 10.1186/s12889-021-11283-w (PMC8223313; doi:10.1186/s12889-021-11283-w)
Supplement: Supplementary file 1 — Additional file 1 Supplementary Table 1. Surveys Description. Supplementary Table 2. Questions used to collect data about the number of days use in the past 30 days for each of the three national health surveys. Supplementary Table 3. List of Mutually Exclusive Categories of single, dual and poly tobacco product use. Supplementary Table 4. Population prevalence of exclusive, dual, and polytobacco users using three frequency of use thresholds for three national surveys*. [file 12889_2021_11283_MOESM1_ESM.docx]

**Supplementary Appendix**

**Supplementary Table 1. Surveys Description**

| **Characteristics** | **Tobacco Use Supplement to the Current Population Survey (TUS-CPS): 2014-2015** | **National Health Interview Survey (NHIS): 2015** | **Population Assessment of Tobacco & Health (PATH): Wave 2; 2014-2015** |
| --- | --- | --- | --- |
| Description | Studies tobacco use behaviors, attitudes, and policies in the United States. | Capture health information and behaviors. Monitors the population´s health towards achieving national health objectives. | Studies tobacco use patterns and health, and produces estimates to inform and assess FDA tobacco regulatory activities |
| Time | Three non-consecutive months every 3 to 4 years as part of the monthly CPS survey | Annually | Annually |
| Total Survey Sample size | 163,920 (total) 157,535 (restricted to first observation of participants) inidividuals for ages 18 and above | 33,672 inidividuals for ages 18 and above | 28,148 inidividuals for ages 18 and above |
| Response rate (formula) | July 2014: 53.7% Jan 2015: 55.7% May 2015: 53.1% (percentage of self-respondents of all eligible survey sample) | Conditional response rate for the Sample Adult component: 79.7%, (calculated by dividing the number of completed Sample Adult interviews (33,672) by the total number of eligible sample adults (42,270); Unconditional or final response rate for the Sample Adult component: 55.2% (calculated by multiplying the conditional rate of 79.7% by the final family response rate 69.3%) | 78.4% (ratio of number of completed cases to number of cases eligible for Wave 2 interview among Wave 1 participants) |
| Sampling frame | National two-stage sampling design of civilian, noninstitutionalized US population | National multistage stratified clustered sampling design of civilian, noninstitutionalized US population; Oversampled Black, Hispanic, and Asian persons | National four-stage stratified area probability design of civilian, noninstitutionalized US population; Oversampled tobacco users, young adults (aged 18-24), and African American persons |
| Data collection method | Two-thirds by telephone and a third through face to face interviews using  Computer Assisted Telephone Interviewing (CATI) or Computer Assisted Personal Interviewing (CAPI) | Face-to-face interviews using  Computer Assisted Personal Interviewing (CAPI) | Audio Computer-Assisted Self-Interviewing (ACASI) or and Computer-Assisted Personal Interviewing (CAPI).  ACASI was used to collect tobacco related information. |

**Supplementary Table 2. Questions used to collect data about the number of days use in the past 30 days for each of the three national health surveys.**

| **Products** | **Tobacco Use Supplement to the Current Population Survey (TUS-CPS)**  **2014-2015** | **National Health Interview Survey (NHIS) 2015** | **Population Assessment of Tobacco & Health (PATH)**  **Wave 2; 2014-2015** |
| --- | --- | --- | --- |
| Cigarettes | (1) "Have you smoked at least 100 cigarettes in your entire life?” If answer “yes”; then (2) “Do you now smoke cigarettes every day, some days or not at all? If answer “some days”; then (3) “On how many of the past 30 days did you smoke cigarettes?" | (1) “Have you smoked at least 100 cigarettes in your entire life?”; then (2) “Do you now smoke every day, some days, or not at all?” those who responded, “every day” or “some days”; and then (3) “On how many of the past 30 days did you smoke a cigarettes?” | Current smoker users who were someday established (>= 100 cigarettes smoked in entire life), current experimental or non-current 30-day smokers were asked “On how many days of the past 30 days did you smoke cigarettes?”. Adult respondents who have ever smoked the product regularly, and currently smokes every day were classified as every day users and coded as 30 days in the past 30 days. |
| Other combustibles | Products were grouped in: 1) regular cigar, cigarillo or little filtered cigar; 2) regular pipe filled with tobacco; 3) water pipe or hookah. Questions. (1) “Have you ever used any of the following [ tobacco products] even one time?”; then (2) “Do you now smoke [the product(s)] every day, some days or not at all?”. If answer “some days” then (3) “On how many of the past 30 days did you smoke [the product(s)]?” | Products were grouped in: 1) cigars (i.e., regular cigar, cigarillo or a little filtered cigar), and 2) pipes (i.e., regular pipe, water pipe or hookah). Questions (1) “Have you ever used the product even one time?”; Then only for cigars (2) “Have you smoked at least a minimum threshold (50 cigars)?; as a follow-up question for cigars and pipes (3) “Do you now smoke the product every day some days or not at all?; then only for cigars (4) “On how many of the past 30 days have you smoked the product?” | Products were grouped in: 1) cigarillos 2) filtered cigars and 3) traditional cigars, 4) pipes, 5) hookah.  Adults that were everyday users, some day users, experimental current users and former thirty day users were asked " On how many days of the past 30-days did you use an [tobacco product]?” |
| Smokeless tobacco | Products were grouped in: 1) smokeless tobacco (i.e., snuff, dip, spit, chew tobacco or snus) and 2) dissolvable tobacco. Questions. (1) “Have you ever used any of the following [tobacco products] even one time?”; then (2) “Do you now use [the product(s)] every day, some days or not at all?”. If answer “some days” then as a follow up questions (3) “On how many of the past 30 days did you use [the product(s)]?”. | Products were grouped in: smokeless tobacco (i.e., chewing tobacco, snuff, dip, snus or dissolvable tobacco). Questions (1) “Have you ever used the product even one time?”; then (2) “Have you used at least a minimum threshold (used smokeless tobacco at least 20 times)?; as a follow-up question (3) “Do you now use the product every day some days or not at all?; and (4) “On how many of the past 30 days have you used the product?” | Products were grouped in: 1) smokeless tobacco (i.e., chewing tobacco, loose snus, moist snuff, dip or spit), 2) snuns and 3) dissolvable tobacco.  Adults that were some day established, current experimental or non-current 30-day users were asked "On how many days of the past 30-days did you use an [tobacco product]?” Adult respondents who have ever smoked the product regularly, and currently smokes every day were classified as every day users and coded as 30 days in the past 30 days. |
| E-cigarettes | Products were grouped in: E-cigarettes (i.e., e-cigarettes, vape-pens, hookah pens, e-hookahs or e-vaporizers) Questions (1) “Have you ever used any of the following [other nicotine products] even one time?”; then (2) “Do you now use [the product(s)] every day, some days or not at all?”. If answer “some days” then as a follow up questions (3) “On how many of the past 30 days did you use [the product(s)]?”. | Products were grouped in: e-cigarettes (i.e., vape-pens, hookah-pens, e-hookahs or e-vaporizers. Questions (1) “Have you ever used the product even one time?”; then (2) “Do you now use the product every day some days or not at all?; and (3) “On how many of the past 30 days have you used the product?” | Products were grouped in: 1) e-cigarettes and 2) electronic nicotine products (i.e., e-cigars, e-pipes, e-hookahs). Adults that were everyday users, some day users, experimental current users and former thirty day users were asked " On how many days of the past 30-days did you use an [e-cigarettes/Epods]?” . |

**Supplementary Table 3: List of Mutually Exclusive Categories of single, dual and poly tobacco product use**

| \| **No use** \| 1. No use any product groups \| \| --- \| --- \| \| **Single tobacco product use** \| 2. Cigarettes  3. E-cigarettes  4. Other combustibles  5. Smokeless \| \| **Dual tobacco product use** \| 6. Cigarettes + e-cigarettes  7. Cigarettes + other combustibles  8. Cigarettes + smokeless  9. E-cigarettes + other combustibles  10. E-cigarettes + smokeless  11. Other combustibles + smokeless \| \| **Polytobacco product use** \| 12. Cigarettes + e-cigarettes + other combustibles  13. Cigarettes + e-cigarettes + smokeless  14. Cigarettes + other combustibles + smokeless  15. E-cigarettes + other combustibles + smokeless  16. All four tobacco product groups \| |
| --- | --- | --- | --- | --- | --- | --- | --- | --- |

**Supplementary Table 4. Population prevalence of exclusive, dual, and polytobacco users using three frequency of use thresholds for three national surveys.***

| **Products** | **TUS-CPS**  **% (95% CI)**^‡^ | | | **NHIS**  **% (95% CI)**^‡^ | | | **PATH**  **% (95% CI)**^‡^ | | |
| --- | --- | --- | --- | --- | --- | --- | --- | --- | --- |
|  | **1+ day** | **10+days** | **25+ days** | **1+ day** | **10+ days** | **25+ days** | **1+ day** | **10+ days** | **25+ days** |
| **Exclusive Use** |  |  |  |  |  |  |  |  |  |
| Cigarettes | 11.1 (10.9 ,11.2) | 11.4 (11.2 ,11.6) | 10.3 (10.1 ,10.4) | 11.7 (11.2 ,12.2) | 11.9 (11.4 ,12.5) | 10.8 (10.3 ,11.4) | 13.1 (12.6 ,13.6) | 14.5 (14.0 ,15.0) | 13.7 (13.2 ,14.2) |
| E-cigarettes | 0.7  (0.6 ,0.7) | 0.6 (0.6 ,0.7) | 0.6 (0.5 ,0.6) | 1.3 (1.1 ,1.4) | 1.0 (0.9 ,1.2) | 0.9 (0.8 ,1.0) | 1.3 (1.2 ,1.4) | 1.2 (1.1 ,1.4) | 1.2 (1.1 ,1.4) |
| Other combustibles | 1.2  (1.2 ,1.3) | 0.4 (0.4 ,0.5) | 0.3 (0.3 ,0.3) | 1.8 (1.6 ,2.1) | 1.2 (1.0 ,1.4) | 1.2 (1.0 ,1.3) | 2.6 (0.0 ,0.0) | 0.8 (0.7 ,0.9) | 0.5 (0.5 ,0.6) |
| Smokeless tobacco | 1.2  (1.1 ,1.2) | 1.1 (1.1 ,1.2) | 1.0 (0.9 ,1.0) | 1.4 (1.2 ,1.7) | 1.4 (1.2 ,1.6) | 1.2 (1.0 ,1.5) | 1.7 (1.5 ,1.9) | 1.7 (1.5 ,1.9) | 1.6 (1.4 ,1.7) |
| **Dual Use** |  |  |  |  |  |  |  |  |  |
| C + EC | 1.2  (1.2 ,1.3) | 0.7 (0.6 ,0.7) | 0.3 (0.3 ,0.3) | 1.4 (1.3 ,1.6) | 0.8 (0.7 ,1.0) | 0.3 (0.3 ,0.5) | 2.2 (2.0 ,2.4) | 1.1 (1.0 ,1.2) | 0.5 (0.5 ,0.6) |
| C + OC | 0.6  (0.5 ,0.6) | 0.2 (0.2 ,0.2) | 0.1 (0.1 ,0.2) | 0.9 (0.8 ,1.0) | 0.6 (0.5 ,0.7) | 0.5 (0.4 ,0.6) | 1.9 (1.7 ,2.0) | 0.7 (0.6 ,0.7) | 0.4 (0.4 ,0.5) |
| C + ST | 0.2  (0.2 ,0.2) | 0.2 (0.1 ,0.2) | 0.1 (0.1 ,0.1) | 0.4 (0.3 ,0.5) | 0.2 (0.2 ,0.3) | 0.1 (0.1 ,0.2) | 0.5 (0.5 ,0.6) | 0.4 (0.3 ,0.4) | 0.2 (0.1 ,0.3) |
| EC + OC | 0.1  (0.1 ,0.1) | 0.01 (0.0 ,0.0) | 0.01 (0.0 ,0.0) | 0.1 (0.0 ,0.1) | 0.04 (0.0 ,0.1) | 0.02 (0.0 ,0.0) | 0.3 (0.2 ,0.4) | 0.03 (0.0 ,0.0) | 0.01 (0.0 ,0.0) |
| EC + ST | 0.03 (0.0 ,0.0) | 0.02 (0.0 ,0.0) | <0.01 (0.0 ,0.0) | 0.04 (0.0 ,0.1) | 0.02 (0.0 ,0.1) | <0.01 (0.0 ,0.0) | 0.04 (0.0 ,0.1) | 0.04 (0.0 ,0.1) | 0.02 (0.0 ,0.0) |
| OC + ST | 0.1  (0.1 ,0.1) | 0.01 (0.0 ,0.0) | <0.01 (0.0 ,0.0) | 0.1 (0.1 ,0.2) | 0.03 (0.0 ,0.1) | 0.02 (0.0 ,0.0) | 0.1 (0.1 ,0.2) | 0.02 (0.0 ,0.0) | 0.01 (0.0 ,0.0) |
| **Poly Use** |  |  |  |  |  |  |  |  |  |
| C+ EC+ OC | 0.11 (0.1 ,0.1) | 0.02 (0.0 ,0.0) | <0.01 (0.0 ,0.0) | 0.23 (0.2 ,0.3) | 0.09 (0.0 ,0.2) | 0.01 (0.0 ,0.1) | 0.51 (0.4 ,0.6) | 0.06 (0.0 ,0.1) | 0.01 (0.0 ,0.0) |
| C + EC+ ST | 0.04 (0.0 ,0.1) | 0.01 (0.0 ,0.0) | <0.01 (0.0 ,0.0) | 0.04 (0.0 ,0.1) | 0.01 (0.0 ,0.0) | NA | 0.13 (0.1 ,0.2) | 0.02 (0.0 ,0.0) | 0.01 (0.0 ,0.0) |
| C+ OC+ ST | 0.04 (0.0 ,0.1) | 0.01 (0.0 ,0.0) | <0.01 (0.0 ,0.0) | 0.06 (0.0 ,0.1) | 0.01 (0.0 ,0.0) | <0.01 (0.0 ,0.0) | 0.15 (0.1 ,0.2) | 0.03 (0.0 ,0.0) | 0.02 (0.0 ,0.0) |
| EC+ OC + ST | <0.01 (0.0 ,0.0) | <0.01 (0.0 ,0.0) | NA | <0.01 (0.0 ,0.0) | NA | NA | 0.03 (0.0 ,0.1) | 0.01 (0.0 ,0.0) | 0.01 (0.0 ,0.0) |
| C+ EC+ OC+ST | 0.02 (0.0 ,0.0) | <0.01 (0.0 ,0.0) | <0.01 (0.0,0.0) | 0.03 (0.0 ,0.1) | <0.01 (0.0 ,0.0) | NA | 0.10 (0.1 ,0.1) | 0.01 (0.0 ,0.0) | <0.01 (0.0 ,0.0) |

Abbreviations: C= cigarettes; EC: e-cigarettes; OC: other-combustibles; ST = smokeless tobacco; TUS-CPS=Tobacco Use Supplement to the Current Population Survey; NHIS=National Health Interview Survey; PATH=Population Assessment of Tobacco and Health.

NA= no sample was available for this group. ‡ 0.0 values in 95% CI are ≤0.01

*Proportions of each product use behavior as a percentage of any users (main text tables) can be estimates as= prevalence of each product use pattern / ∑ of all prevalence for that product
